# Clinical and Epidemiological Factors Associated with Methicillin Resistance in Community-Onset Invasive *Staphylococcus aureus* Infections: Prospective Multicenter Cross-Sectional Study in Korea

**DOI:** 10.1371/journal.pone.0114127

**Published:** 2014-12-08

**Authors:** Eu Suk Kim, Hong Bin Kim, Gayeon Kim, Kye-Hyung Kim, Kyung-Hwa Park, Shinwon Lee, Young Hwa Choi, Jongyoun Yi, Chung Jong Kim, Kyoung-Ho Song, Pyoeng Gyun Choe, Nam-Joong Kim, Yeong-Seon Lee, Myoung-don Oh, and the Korea INfectious Diseases (KIND) study group

**Affiliations:** 1 Department of Internal Medicine, Seoul National University Bundang Hospital, Seongnam, Republic of Korea; 2 Department of Internal Medicine, Seoul National University College of Medicine, Seoul, Republic of Korea; 3 Department of Internal Medicine, Seoul National University Hospital, Seoul, Republic of Korea; 4 Department of Internal Medicine, Pusan National University School of Medicine, Yangsan, Republic of Korea; 5 Department of Internal Medicine, Chonnam National University Medical School, Gwangju, Korea; 6 Department of Internal Medicine, Daegu Fatima Hospital, Daegu, Republic of Korea; 7 Department of Infectious Diseases, Ajou University School of Medicine, Suwon, Republic of Korea; 8 Department of Laboratory Medicine, Pusan National University School of Medicine, Yangsan, Republic of Korea; 9 Center for Infectious Diseases, Korea National Institute of Health, Osong, Republic of Korea; Rockefeller University, United States of America

## Abstract

Successful empirical therapy of *Staphylococcus aureus* infections requires the ability to predict methicillin resistance. Our aim was to identify predictors of methicillin resistance in community-onset (CO) invasive *S. aureus* infections. Sixteen hospitals across Korea participated in this study from May to December 2012. We prospectively included cases of *S. aureus* infection in which *S. aureus* was isolated from sterile clinical specimens ≤72 hours after hospitalization. Clinical and epidemiological data were gathered and compared in methicillin-resistant *S. aureus* (MRSA) and methicillin-susceptible *S. aureus* (MSSA) cases. Community-associated (CA) infections were defined as in previous studies. In total, there were 786 cases of community-onset *S. aureus* infection, 102 (13.0%) of which were CA-MRSA. In addition to known risk factors, exposure to 3rd generation cephalosporins in the past 6 months [odds ratio (OR), 1.922; 95% confidence interval (CI), 1.176–3.142] and close contact with chronically ill patients in the past month (OR, 2.647; 95% CI, 1.189–5.891) were independent risk factors for MRSA infection. However, no clinical predictors of CA-MRSA were identified. Methicillin resistance, CO infection, and appropriateness of empirical antibiotics were not significantly related to 30-day mortality. MRSA infection should be suspected in patients recently exposed to 3rd generation cephalosporins or chronically-ill patients. There were no reliable predictors of CA-MRSA infection, and mortality was not affected by methicillin resistance.

## Introduction

Infections caused by methicillin-resistant *Staphylococcus aureus* (MRSA) have been reported worldwide in individuals without apparent healthcare-associated (HA) risk factors [1]. The term “community-associated MRSA” (CA-MRSA) has been coined to distinguish the clinical and microbiological features of these infections from traditional HA-MRSA [2]. CA-MRSA was the most common identifiable cause of skin and soft tissue infections (SSTIs) among patients treated in US emergency rooms, and most clones were of the USA300 pulsed-field type containing Panton-Valentine leucocidin (PVL) [3]. However, the prevalence and characteristics of CA-MRSA vary with geographical location [4].

Although MRSA is endemic in Korean hospitals, cases of CA-MRSA have not been common [5]. In studies to date, the most prevalent CA-MRSA clone was sequence type 72, staphylococcal chromosomal cassette *mec* type IVa (ST72-SCC*me*c IVa) without the PVL gene [5], [6]. However, this clone is likely to have become more common in recent years, not only in the community but also in hospitals [7], [8], but there has been no large scale multicenter study on the epidemiological changes of CA-MRSA infections in Korea since 2005.

The ability to predict methicillin resistance in invasive community-onset (CO) *S. aureus* infections is necessary for successful empirical treatment. The Centers for Disease Control and Prevention (CDC) in the USA has defined the clinical criteria for risk factors associated with MRSA acquisition to distinguish CA-MRSA from HA-MRSA [9], and these can guide clinicians in their initial choice of empirical antibiotic in cases of suspected invasive MRSA infections. However, it is not clear whether empirical anti-MRSA drugs should be considered in suspected cases of invasive CO-*S. aureus* infections in Korea. It may be difficult to distinguish cases of CA-MRSA without recognizable risk factors from CA-MSSA. No predictors of CA-MRSA infection have been clearly identified in previous prospective studies, all of which were single-center [10]–[12].

This multicenter nationwide cross-sectional study was performed to evaluate epidemiological changes in invasive CA-MRSA infections in Korea. We also tried to identify clinical and epidemiological risk factors for methicillin resistance in patients with invasive CO-*S. aureus* infections, which are important for more rational anti-MRSA antibiotic use.

## Materials and Methods

### Study design and population

Sixteen hospitals across Korea participated in this eight-month study from May to December 2012. To identify cases of invasive *S. aureus* infection, infectious diseases specialists at each hospital regularly screened microbiological cultures that would normally be expected to be sterile for the presence of *S. aureus*. Patients were enrolled if their culture was positive for *S. aureus*, and samples for culture were collected within 72 hours of hospitalization. If a patient had more than one invasive *S. aureus* infection during the study period, only the first incident was included as a study case. Patients who were discharged from the hospital or who died before it was possible to confirm *S. aureus* infection microbiologically were excluded from the analysis. The study was approved by the Institutional Review Board at each participating hospital: All participating institutions were in the Republic of Korea. 1) Seoul National University Bundang Hospital Institutional Review Board, Seongnam, 2) Seoul National University Hospital Institutional Review Board, Seoul, 3) Pusan National University Hospital Institutional Review Board, Busan, 4) Chonnam National University Hospital Institutional Review Board, Gwangju, 5) Daegu Fatima Hospital Institutional Review Board, Daegu, 6) Ajou University Hospital Institutional Review Board, Suwon, 7) Institutional Review Board of Inje University Busan Paik Hospital, Busan, 8) Boramae Hospital Institutional Review Board, Seoul, 9) Ewha Womans University Mokdong Hospital Institutional Review Board, Seoul, 10) Soonchunhyang University Seoul Hospital Institutional Review Board, Seoul, 11) Soonchunhyang University Bucheon Hospital Institutional Review Board, Bucheon, 12) Yonsei University Wonju Severance Christian Hospital Institutional Review Board, Wonju, 13) Soonchunhyang University Cheonan Hospital Institutional Review Board, Cheonan, 14) Institutional Review Board of Chonbuk National University Hospital, Jeonju, 15) Chonnam National University Hwasun Hospital Institutional Review Board, Hwasun, 16) Pusan National University Yangsan Hospital Institutional Review Board, Yangsan. The informed consents were waived.

### Definitions

Isolation of *S. aureus* and antibiotic susceptibility testing were performed in the clinical microbiology laboratory of each participating hospital using an automated system. Invasive *S. aureus* infection was diagnosed when *S. aureus* was isolated from a normally sterile body fluid, as defined previously [13]. The infection was classified as community-onset (CO) if the *S. aureus*-positive specimen was obtained within 72 hours of hospitalization. It was classified as community-associated (CA) if the specimen was not from a site of previous surgery, and none of the established healthcare-associated (HA) risk factors for MRSA acquisition or infection were present [9], [11], [14]. These included: (1) history of hospitalization (excluding birth of normal newborns) or surgery within 1 year of the *S. aureus*-positive culture; (2) history of residence in a long-term care facility within the past year; (3) history of hemodialysis or peritoneal dialysis within the past year; and (4) presence of a permanent indwelling catheter or percutaneous medical device at the time of culture. The cases that did not fully meet these criteria were classified as CO-HA *S. aureus* infections.

In each case, the type of *S. aureus* infection was confirmed clinically by an infectious diseases specialist at each hospital and classified as one of the following: central line-associated bloodstream infection (CLA-BSI), pneumonia, SSTI, surgical site infection (SSI), bone and joint infection (BJI), otitis, endocarditis, intra-abdominal infection (IAI), urinary tract infection (UTI), central nervous system (CNS) infection, vascular infection including arteriovenous fistula (AVF) infection, and bacteremia of unknown primary focus. Cases of pneumonia, SSTI, UTI, and otitis were included only when *S. aureus* was isolated from sterile body fluids such as blood or surgical specimen. Antibiotic therapy was considered appropriate if the isolate was susceptible to the chosen antibiotic in vitro and the treatment was administered for at least 24 hours within 72 hours of hospitalization.

### Data collection

All data were collected prospectively. Infectious diseases specialists at each hospital reviewed the patients' medical records and interviewed the enrolled patients using a standardized clinical record form. Among the data collected were (1) demographic findings, (2) clinical characteristics, including the Charlson comorbidity index [15] and 30-day mortality, and (3) microbiological data. We also gathered diverse epidemiological data to assess new potential risk factors for MRSA infection.

### Statistical analysis

We assumed the prevalence of any risk factor for CA-MRSA to be 15%, and the difference in prevalence between CA-MRSA and CA-MSSA to be 10%. We also hypothesized that the ratio of CA-MRSA to CA-MSSA would be 1∶2. To achieve a 0.80 level of statistical power with a two-sided alpha error of 0.05, we estimated that the study should last at least 8 months to allow the 16 hospitals to enroll a total of 296 cases of CA-*S. aureus* infection. The sample size was calculated using Epi Info version 7 developed by the CDC and the estimate of study duration was based on the number of invasive *S. aureus* cases recorded at each hospital during the previous year.

Statistical analyses were performed with PASW Statistics, version 18.0.0. Univariate analyses were performed to screen for potential risk factors (using Student's t-test and the χ2 test or Fisher's exact test, depending on the type of variable), with *P* values <0.10 considered statistically significant for inclusion in the multivariate analysis. Multivariate linear logistic regression models were formulated and tested to adjust for covariates. Adjusted odds ratios (ORs) and their 95% confidence intervals (CIs) were calculated, with *P* values <0.05 considered statistically significant.

## Results

### Surveillance

**Figure 1:**
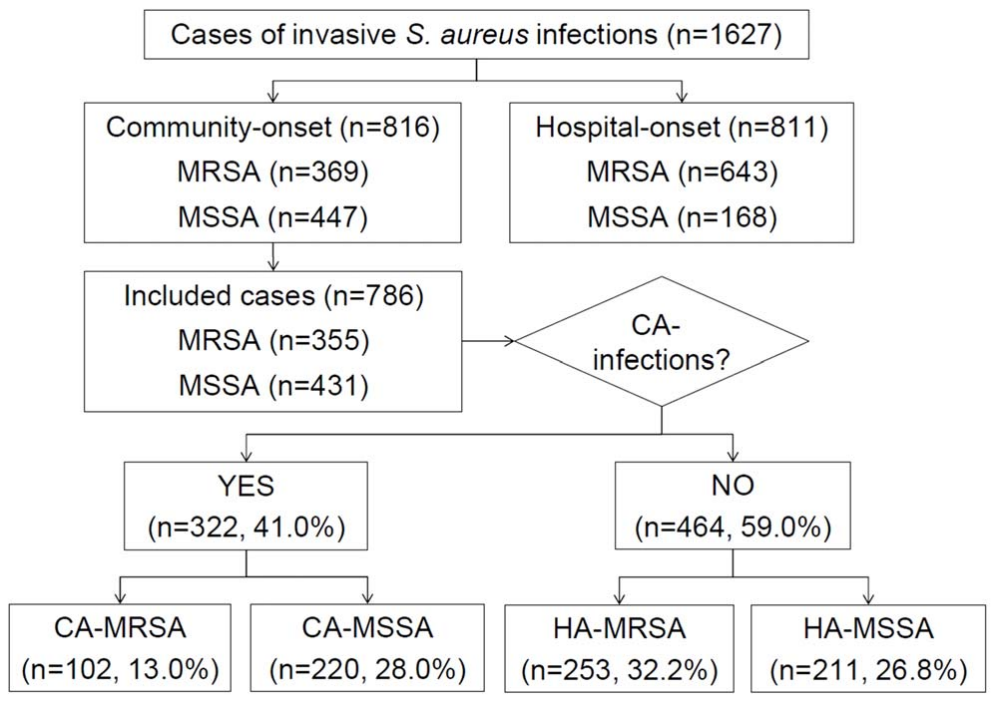
Schematic diagram describing the classification of *Staphylococcus aureus* infections as community-associated (CA) or healthcare-associated (HA) according to methicillin-resistance (MRSA) or methicillin susceptibility (MSSA).

The 16 participating hospitals are spread across the country. All are teaching hospitals; 15 (93.8%) are university-affiliated, and 8 (50.0%) have 900 or more beds. Two of the hospitals participated for 6 months only, from May to October 2012. A total of 1627 cases of invasive *S. aureus* infection were recorded during the study period, and 786 cases of CO-*S. aureus* infection were included in the analysis after excluding 811 hospital onset cases and 30 cases in which patients were discharged or died before microbiological confirmation of *S. aureus* infection (Fig. 1). Among the 786 cases included, 322 cases (41.0%) were CA infections and 464 (59.0%) were HA infections. The most common specimen from which *S. aureus* was isolated was blood (68.3%). Other specimen types were abscesses in internal body sites (13.4%), bone and organ tissue (6.7%), joint fluid (5.5%), ear discharge (1.8%), pleural fluid (1.7%), ascites (1.3%), CSF (1.3%), and pericardial fluid (0.1%).

### Clinical characteristics and antibiotic susceptibility

The clinical characteristics of the 786 patients with CO-*S. aureus* infections are summarized in Table 1. The patients were older and there were more comorbidities among the HA infection group than among the CA group. The most common infections were SSTI in the CA-MRSA group, BJI in the CA-MSSA group, and pneumonia in the HA-MRSA group.

**Table 1:** Baseline characteristics of 786 cases of community-onset invasive *Staphylococcus aureus* infection in Korea according to epidemiological features and methicillin resistance.

Characteristics	CA-Infections		HA-Infections	
	CA-MRSA (n = 102)	CA-MSSA (n = 220)	HA-MRSA (n = 253)	HA-MSSA (n = 211)
Sex (M:F)	61:41	135:85	151:102	130:81
Age, years (mean ± SD)	46.2±26.2	49.1±26.4	61.2±21.2	59.2±19.4
Range	0–89	0–94	0–100	0–100
Type of infection				
Primary bacteremia	14 (13.7%)	39 (17.7%)	32 (12.6%)	27 (12.8%)
CLA-BSI	0 (0%)	0 (0%)	20 (7.9%)	20 (9.5%)
Pneumonia	4 (3.9%)	17 (7.7%)	51 (20.2%)	20 (9.5%)
SSTI	34 (33.3%)	52 (23.6%)	30 (11.9%)	43 (20.4%)
SSI	0 (0%)	0 (0%)	26 (10.3%)	18 (8.5%)
BJI	19 (18.6%)	72 (32.7%)	39 (15.4%)	44 (20.9%)
Otitis	14 (13.7%)	11 (5.0%)	3 (1.2%)	0 (0%)
Endocarditis	5 (4.9%)	12 (5.5%)	5 (2.0%)	3 (1.4%)
IAI	4 (3.9%)	9 (4.1%)	22 (8.7%)	20 (9.5%)
UTI	4 (3.9%)	5 (2.3%)	6 (2.4%)	4 (1.9%)
CNS infection	4 (3.9%)	3 (1.4%)	4 (1.6%)	3 (1.4%)
Vascular infection	0 (0%)	0 (0%)	15 (5.9%)	8 (3.8%)
Others	0 (0%)	0 (0%)	0 (0%)	1 (0.5%)
Comorbidities				
None	72 (70.6%)	135 (61.4%)	49 (19.4%)	43 (20.4%)
Charlson index (mean ± SD)	0.5±1.2	0.7±1.2	2.5±2.2	2.5±.3

CA, community-associated; HA, healthcare-associated; MRSA, methicillin-resistant *S. aureus*; MSSA, methicillin-susceptible *S. aureus*; CLA-BSI, central line associated blood stream infection; SSTI, skin and soft tissue infection; SSI, surgical site infection; BJI, bone and joint infection; IAI, intra-abdominal infection; UTI, urinary tract infection; CNS, central nervous system.

**Figure 2:**
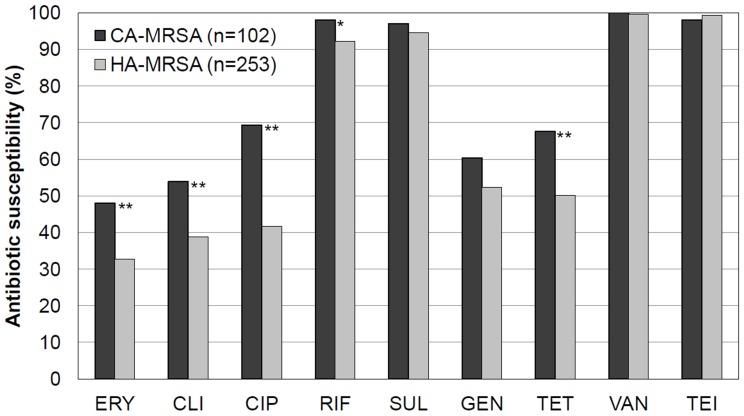
Antibiotic susceptibility profiles of the community-associated methicillin-resistant *Staphylococcus aureus* (CA-MRSA) isolates and the healthcare-associated MRSA (HA-MRSA) isolates. ERY, CLI, CIP, RIF, SUL, GEN, TET, VAN, and TEI denote erythromycin, clindamycin, ciprofloxacin, rifampin, sulfamethoxazole-trimethoprim, gentamicin, tetracycline, vancomycin, and teicoplanin, respectively. Data for CIP, RIF, SUL, GEN, TET, and TEI were missing for 2, 1, 34, 4, 2, and 1 patient(s), respectively. * and ** indicate statistical significance at *P*<0.05 and 0.01, respectively (χ2 test).

A comparison of their antibiotic susceptibility profiles showed that the CA-MRSA isolates (n = 102) were more susceptible to non-beta-lactam agents such as erythromycin, clindamycin, ciprofloxacin, rifampin, and tetracycline than the HA-MRSA isolates (n = 253), but the same was not true for sulfamethoxazole-trimethprim and gentamicin (Fig. 2).

### Risk factors for methicillin resistance

Among the 786 invasive CO-*S. aureus* infections, 431 were caused by MSSA and 355 by MRSA. The risk factors for methicillin resistance identified in univariate analysis are shown in Table 2. According to our multivariate logistic regression model, factors associated with an increased risk of invasive CO-MRSA infection included residence in a long-term care facility within the past year, history of SSI within the past year, history of MRSA carriage within the past year, recent (in the past 6 months) exposure to 3rd generation cephalosporins, and history of close contact with patients with chronic illnesses within the past month when compared to CO-MSSA infection (Table 3).

**Table 2:** Univariate analysis of risk factors for methicillin resistance in 786 cases of community-onset invasive *Staphylococcus aureus* infection.

Characteristics	MSSA (n = 431)	MRSA (n = 355)	*P* Value	OR (95% CI)
Male gender	265 (61.5%)	212 (59.7%)	0.614	1.077 (0.808–1.435)
Age, years (mean ± SD)	54.0±23.8	56.9±23.7	0.091	
Age (per 10-year increase)			0.091	**1.053 (0.992**–**1.118)**
Age group, years			0.074	
≤15	49 (11.4%)	38 (10.7%)		
16-64	207 (48.0%)	145 (40.8%)		
≥65	175 (40.6%)	172 (48.5%)		
Previous admission (<1 yr)	171 (39.7%)	204 (57.5%)	<0.001	**2.054 (1.544**–**2.733)**
Residence in a long -term care facility (<1 yr)	20 (4.6%)	44 (12.4%)	<0.001	**2.907 (1.680**–**5.033)**
History of dialysis (<1 yr)	34 (7.9%)	43 (12.1%)	0.047	**1.609 (1.002**–**2.584)**
History of SSI (<1 m)	11 (2.6%)	23 (6.5%)	0.007	**2.654 (1.271**–**5.504)**
Presence of percutaneous devices	37 (8.6%)	50 (14.1%)	0.014	**1.746 (1.112**–**2.739)**
Underlying illness	253 (58.7%)	234 (65.9%)	0.038	**1.361 (1.017**–**1.821)**
Charlson index (mean ± SD)	1.6±2.0	1.9±2.1	0.015	
Charlson index (per 1 unit increase)			0.016	**1.087 (1.016**–**1.164)**
Previous MRSA carriage (<1 yr)	8 (1.9%)	46 (13.0%)	<0.001	**7.871 (3.663**–**16.915)**
Previous antibiotic exposure (<6 m)				
Any antibiotic	109 (25.3%)	161 (45.4%)	0.001	**2.452 (1.814**–**3.314)**
Any cephalosporins	61 (14.2%)	95 (26.8%)	0.001	**2.216 (1.548**–**3.172)**
1st generation cephalosporins	19 (4.4%)	21 (5.9%)	0.339	1.363 (0.721–2.578)
2nd generation cephalosporins	5 (1.2%)	5 (1.4%)	0.761	1.217 (0.350–4.238)
Cephamycin	4 (0.9%)	6 (1.7%)	0.360	1.835 (0.514–6.555)
3rd generation cephalosporins	33 (7.7%)	68 (19.2%)	<0.001	**2.858 (1.836**–**4.449)**
4th generation cephalosporins	5 (1.2%)	4 (1.1%)	1.000	0.971 (0.259–3.643)
Quinolones	16 (3.7%)	35 (9.9%)	<0.001	**2.837 (1.543**–**5.217)**
Immunosuppressant use (<1 yr)	26 (6.0%)	20 (5.6%)	0.813	0.930 (0.510–1.696)
Steroid use	8 (1.9%)	9 (2.5%)	0.515	1.375 (0.525–3.602)
History of acupuncture (<1 m)	26 (6.0%)	17 (4.8%)	0.445	0.783 (0.418–1.468)
History of OPD visit (<1 yr)^a^				
Any number of visits	285 (66.7%)	260 (74.1%)	0.026	**1.424 (1.042**–**1.945)**
1–4 visits/yr	90 (31.6%)	96 (36.9%)		
5–9 visits/yr	60 (21.1%)	47 (18.1%)		
10–19 visits/yr	64 (22.5%)	51 (19.6%)		
20–29 visits/yr	31 (10.9%)	25 (9.6%)		
30–49 visits/yr	7 (2.5%)	6 (2.3%)		
>49 visits/yr	5 (1.8%)	15 (5.8%)		
Unknown	28 (9.8%)	20 (7.7%)		
Close contact with HCWs (<1 m)	111 (25.8%)	135 (38.0%)	<0.001	**1.769 (1.305**–**2.398)**
Close contact with chronically-ill patients (<1 m)	10 (2.3%)	24 (6.8%)	0.002	**3.053 (1.440**–**6.473)**
Living with children ≤5 years old^b^	66 (23.2%)	64 (25.9%)	0.461	1.160 (0.781–1.724)
Current smoking	54 (12.5%)	32 (9.0%)	0.116	0.692 (0.436–1.098)
Alcohol intake^c^	94 (22.3%)	57 (16.7%)	0.051	**0.696 (0.483**–**1.003)**
None	327 (77.7%)	285 (83.3%)		
<20 g/day	38 (9.0%)	23 (6.7%)		
20–40 g/day	15 (3.6%)	11 (3.2%)		
≥40 g/day	41 (9.7%)	23 (6.7%)		
Communal living	10 (2.3%)	21 (5.9%)	0.010	**2.647 (1.230**–**5.698)**
Participation in extreme sports	10 (2.3%)	3 (0.8%)	0.107	0.359 (0.098–1.314)

Data are numbers (%) of patients unless stated otherwise. Values in bold indicate factors with statistical significance (*P*<0.10). MSSA, methicillin-susceptible *S. aureus*; MRSA, methicillin-resistant *S. aureus*; SD, standard deviation; SSI, surgical site infection; OPD, outpatient department; HCW, healthcare worker.

a Data on OPD visits for 8 patients were censored, and 778 patients (427 and 351 patients in the MSSA and MRSA groups, respectively) were included in the analysis.

b Data on living with children ≤5 years old for 254 patients were censored, and 532 patients (285 and 247 patients in the MSSA and MRSA groups, respectively) were included in the analysis.

c Data on alcohol intake for 23 patients were censored, and 763 patients (421 and 342 patients in the MSSA and MRSA groups, respectively) were included in the analysis.

**Table 3:** Multivariate analysis of risk factors for methicillin-resistant *Staphylococcus aureus* (MRSA) infection in patients with community-onset invasive *S. aureus* infection.

Characteristics	*P Value*	Adjusted OR (95% CI)
Residence in a long-term care facility (<1 yr)	0.008	2.438 (1.258–4.726)
History of surgical site infection (<1 m)	0.022	2.481 (1.138–5.410)
Previous MRSA carriage (<1 yr)	<0.001	5.565 (2.501–12.381)
Previous use of 3rd generation cephalosporins (<6 m)	0.009	1.922 (1.176–3.142)
Close contact with chronically-ill patients (<1 m)	0.017	2.647 (1.189–5.891)

In a univariate analysis of 322 cases of invasive CA-*S. aureus* infections, we compared the risk factors for CA-MRSA and CA-MSSA. While SSTIs and otitis were more common in the patients with CA-MRSA, BJIs were more common in those with CA-MSSA (Table 4). However, no risk factor could differentiate significantly between CA-MRSA and CA-MSSA.

**Table 4:** Univariate analysis of risk factors for methicillin-resistant *Staphylococcus aureus* (MRSA) infection among 322 patients with community-associated invasive *S. aureus* infection in Korea.

Characteristics	CA-MSSA (n = 220)	CA-MRSA (n = 102)	*P* Value	OR (95% CI)
Male gender	135 (61.4%)	61 (59.8%)	0.790	1.068 (0.661–1.725)
Age, years (mean ± SD)	46.2±26.2	49.1±26.4	0.369	
Age (per 10-year increase)			0.368	0.960 (0.879–1.049)
Age group				
≤15 yr	40 (18.2%)	20 (19.6%)	0.879	1.098 (0.604–1.994)
16–64 yr	101 (45.9%)	55 (53.9%)	0.663	1.379 (0.861–2.208)
≥65 yr	79 (35.9%)	27 (26.5%)	0.121	0.643 (0.382–1.080)
Type of infection				
Primary bacteremia	39 (17.7%)	14 (13.7%)	0.460	0.738 (0.381–1.431)
Pneumonia	17 (7.7%)	4 (3.9%)	0.218	0.487 (0.160–1.487)
SSTI	52 (23.6%)	34 (33.3%)	0.090	**1.615 (0.964**–**2.701)**
BJI	72 (32.7%)	19 (18.6%)	0.013	**0.471 (0.265**–**0.834)**
Otitis	11 (5.0%)	14 (13.7%)	0.013	**3.023 (1.321**–**6.919)**
Endocarditis	12 (5.5%)	5 (4.9%)	0.951	0.894 (0.306–2.607)
AI	9 (4.1%)	4 (3.9%)	0.816	0.957 (0.288–3.183)
UTI	5 (2.3%)	4 (3.9%)	0.637	1.755 (0.461–6.678)
CNS infections	3 (1.4%)	4 (3.9%)	0.292	2.952 (0.648–13.443)
Presence of comorbidities	85 (38.6%)	30 (29.4%)	0.108	0.662 (0.399–1.097)
Charlson index (mean ± SD)	1.2±0.7	1.2±0.5	0.211	
Charlson index (per 1 unit increase)			0.214	0.867 (0.693–1.086)
Previous MRSA carriage (<1 yr)	0	2 (2.0%)	0.100	-
Previous antibiotic exposure (<6 m)				
Any	20 (9.1%)	7 (6.9%)	0.502	0.737 (0.301–1.803)
Cephalosporins	5 (2.3%)	5 (4.9%)	0.298	2.216 (0.627–7.934)
Quinolones	1 (0.5%)	0 (0%)	1.000	-
Immunosuppressant use (<1 yr)	5 (2.3%)	0 (0%)	0.183	-
Steroid use	2 (0.9%)	0 (0%)	1.000	-
History of acupuncture (<1 m)	15 (6.8%)	10 (9.8%)	0.352	1.486 (0.643–3.431)
History of OPD visit (<1 yr)^a^	121 (55.5%)	65 (65.0%)	0.111	1.489 (0.912–2.430)
Close contact with HCWs (<1 m)	36 (16.4%)	22 (21.6%)	0.258	1.406 (0.778–2.540)
Close contact with chronically-ill patients (<1 m)	3 (1.4%)	2 (2.0%)	0.654	1.447 (0.238–8.794)
Living with children ≤5 years old^b^	36 (24.3%)	16 (27.6%)	0.628	1.185 (0.596–2.357)
Current smoking	34 (15.5%)	11 (10.8%)	0.261	0.661 (0.320–1.365)
Alcohol intake^c^	59 (27.7%)	26 (26.5%)	0.830	0.943 (0.550–1.617)
Communal living	2 (0.9%)	1 (1.0%)	1.000	1.079 (0.097–12.040)
Participation in extreme sports	9 (4.1%)	1 (1.0%)	0.179	0.232 (0.029–1.857)

Data are number (%) of patients unless stated otherwise. Values in bold indicate factors with statistical significance (*P*<0.10). CA, community-associated; MSSA, methicillin-susceptible *S. aureus*; SD, standard deviation; SSTI, skin and soft tissue infection; BJI, bone and joint infection; IAI, intra-abdominal infection; UTI, urinary tract infection; CNS, central nervous system; OPD, outpatient department; HCWs, healthcare workers.

a Data on OPD visits for 4 patients were censored, and 318 patients (218 and 100 patients in the MSSA and MRSA groups, respectively) were included in the analysis.

b Data on living with children ≤5 years old for 116 patients were censored, and 206 patients (148 and 58 patients in the MSSA and MRSA groups, respectively) were included in the analysis.

c Data on alcohol intake for 11 patients were censored, and 311 patients (213 and 98 patients in the MSSA and MRSA groups, respectively) were included in the analysis.

### Clinical outcomes

We were able to assess 30-day mortality in 660 (84.0%) patients, having excluded 126 patients who were transferred or discharged without outpatient follow-up within 30 days of admission. The 30-day mortality rate was 15.3% (101/660) among the patients with invasive CO-*S. aureus* infections. The factors associated with 30-day mortality were older age, higher Charlson comorbidity index, and pneumonia or primary bacteremia (Table 5). However, appropriateness of the initial choice of antibiotic, and resistance of *S. aureus* to methicillin were not significantly associated with mortality. CA infections were significantly associated with lower mortality in the univariate analysis, but not in the multivariate model in CO-*S. aureus* infections.

**Table 5:** Analysis of risk factors for 30-day mortality in community-onset invasive *Staphylococcus aureus* infections in Korea.

Characteristics	Survived (n = 559)	Deceased (n = 101)	OR (95% CI)	Adjusted OR (95% CI)
Male gender	337 (60.3%)	59 (58.4%)	1.081 (0.703–1.662)	
Age, years (mean ± SD)	51.7±24.2	71.8±12.3		
Age (per 10-year increase)			**1.977 (1.640**–**2.384)**	**1.686 (1.345**–**2.112)**
Type of infection				
SSTI	132 (23.6%)	6 (5.9%)	**0.204 (0.088**–**0.477)**	Reference
Pneumonia	41 (7.3%)	35 (34.7%)	**6.700 (3.988**–**11.255)**	**8.674 (3.232**–**23.280)**
BJI	137 (24.5%)	11 (10.9%)	**0.376 (0.196**–**0.725)**	1.859 (0.641–5.390)
Primary bacteremia	62 (11.1%)	31 (30.7%)	**3.550 (2.156**–**5.844)**	**9.274 (3.485**–**24.683)**
CA infections	241 (43.1%)	25 (24.8%)	**0.434 (0.268**–**0.703)**	1.095 (0.556–2.157)
Charlson index (per 1 unit increase)			**1.320 (1.207**–**1.443)**	**1.303 (1.123**–**1.512)**
Appropriate choice of initial antibiotics	410 (73.3%)	66 (65.3%)	0.685 (0.437–1.075)	
Methicillin resistance	252 (45.1%)	51 (50.5%)	1.243 (0.813–1.899)	

Data are number (%) of patients unless stated otherwise. Values in bold indicate factors with statistical significance (*P*<.05). Out of 786 patients, 660 were enrolled in the study, and the outcomes of 126 patients (16.2%) could not be evaluated mainly because of transfer or discharge without follow-up within 30 days of admission. SD, standard deviation; SSTI, skin and soft tissue infection; BJI, bone and joint infection; CA, community-associated.

## Discussion

Of the 1012 cases of invasive MRSA, 102 (10.1%) were caused by CA-MRSA, which is a considerably higher proportion than estimated in previous surveillance studies in Korea (5.3–5.9%) [5. 8]. A similar increase in CA-MRSA has been observed in North America, in both children and adults [16]–[18]. Since the 2000s, CA-MRSA has been the most common identifiable cause of purulent SSTIs in patients treated in US emergency departments [19]. The prescribing practices of clinicians for treating SSTIs in emergency departments have changed as a result, from MRSA-inactive to MRSA-active empirical antimicrobial regimens. An MRSA-targeted choice of empirical antibiotics is not yet common in emergency rooms in Korea because there have been no data supporting this strategy. However, we should be aware that guidance about the appropriate antibiotic usage may become important as numbers of CA-MRSA cases rise.

Previously established risk factors for MRSA acquisition in other studies were also significant in our univariate analysis (Table 2) [9], [11], [14]. However, only three remained significant in the multivariate model: residence in a long-term care facility within the past year, history of SSI within the past month, and history of MRSA carriage within the past year (Table 3). Additionally, exposure to 3rd generation cephalosporins within the past six months and close contact with chronically-ill patients within the past month were found to be significant risk factors for CO-MRSA infection in a multivariate analysis. Recent antibiotic exposure has been identified as a significant risk factor for methicillin resistance in CO-*S. aureus* infections in other studies [12], [20], [21]. Use of fluoroquinolones has been considered a particularly important predictor of nosocomial MRSA infections [22], [23], while exposure to 3rd generation cephalosporins was a risk factor in a case-control study in a Japanese geriatric hospital [24]. Although exposure to broad-spectrum antibiotics was also an independent risk factor in our study, it remains likely that antibiotic use is simply an indicator of exposure to HA environments. Further research is needed to clarify the association of methicillin resistance with exposure to diverse antibiotics in community-onset *S. aureus* infections.

Another predictor of MRSA infection in our study was recent close contact with a chronically-ill patient. Such patients are much more likely than the general population to have been exposed to hospital environments or invasive procedures. In the traditional family model in Korea, the primary caregivers for patients with chronic illnesses are often family members even after hospitalization [25]. Rates of *S. aureus* transmission from patients to household members reportedly range from <10% to 43% [26]. Transmission of CA-MRSA within the family has also been reported [27]. Taken together, growing evidence suggests that family members or primary caregivers for patients with chronic conditions could be at increased risk of acquiring MRSA. It is possible that younger children, with their relatively poor understanding of hygiene and their susceptibility to infections, might easily transmit MRSA in the community, but they are not associated with methicillin resistance in our study.

The Charlson comorbidity index was related to methicillin resistance in univariate analysis but not in multivariate analysis. Other host-related factors such as age or gender were also unrelated to methicillin resistance. Diabetes was found to be a predictive factor for MRSA colonization or infection in some studies [28], [29]. However, it did not differ significantly between MRSA and MSSA infections among either CA- or HA-infections in this study.

Previous studies investigating the predictors of CA-MRSA among patients without any known risk factors for MRSA were performed in single centers and failed to identify reliable and distinguishable factors [10]–[12]. Despite our improved study design (multi-center, large-scale, nationwide prospective study), we also found no significant clinical or epidemiological factors that distinguish between CA-MSSA and CA-MRSA. We cannot rule out the possibility that important factors have been missed. However, it is also possible that identifying distinguishing features of CA-MRSA in Korea may be more difficult than in other populations because the same MRSA clones are frequently present in the hospital setting and in the community [5], [7].

We have in addition compared the variables for HA-MRSA (n = 253) and HA-MSSA (n = 211) infections in the same way as in CA-infections (S1 Table and S2 Table). In multivariate analysis, history of MRSA carriage within the past year, exposure to 3rd generation cephalosporins within 6 months, and history of close contact with patients with chronic illnesses within the past month were statistically significant risk factors for methicillin resistance in CO-HA-*S. aureus* infections, as in the analysis of whole CO-*S. aureus* infections. The only distinguishing factor favoring MRSA infection in this setting was presentation as pneumonia (odds ratio 1.975, 95% confidential interval 1.073–3.633). Two recent studies of the clinical characteristics of infections caused by the ST72-SCC*mec* IV strain, which is the most common genotype of CA-MRSA in Korea, also found that pneumonia was one of the leading diagnoses among CO-MRSA infections [30], [31].

CA infection, methicillin resistance, and appropriateness of initial choice of antibiotic did not seem to affect 30-day mortality in this study. When we stratified the cases as CA-infections or HA-infections, methicillin resistance and appropriateness of antibiotic choice again had no significant effect on 30-day mortality. Instead 30-day mortality was primarily related to patient's status and severity of infection. A recent Austrian study identified higher patient age, pneumonia as the type of infection, and failure to use an MRSA-active antimicrobial agent, as significant risk factors for early mortality from MRSA bacteremia [32]. However, two Taiwanese studies of CA- or CO-*S. aureus* bacteremia showed that 30-day mortality rates were not affected by community origin, methicillin resistance, or the initial choice of empirical antibiotics, which is consistent with our findings [33], [34]. We suggest that clinicians can safely defer prescribing empirical glycopeptides in cases of suspected invasive CO-*S. aureus* infections when no risk factors for MRSA infection are present and bacteremia or pneumonia is not clinically suspected.

This study has potential limitations. First, the number of CA-MRSA cases may have been underestimated because we included only those that were confirmed microbiologically. Because microbiological confirmation is frequently difficult in SSTIs, and most of the CA-MRSA clones in Korea do not produce the PVL toxin, it is possible that some cases of SSTI were missed [5], [6], [35]. It is worth noting, however, that SSTIs comprised 33% of the CA-MRSA infections included (Table 1). This is not only consistent with a previous Korean study [5], but also suggests that the risk of missing SSTIs was in fact low. Second, as we excluded patients who were discharged or died before microbiological confirmation of *S. aureus* infection, a selection bias cannot be ruled out. However, the excluded cases comprised only 3.7% (30/816) of the total, and the rates were similar in the MRSA and MSSA groups. Third, the findings associated with 30-day mortality in our study should be interpreted with caution because we did not include data for those patients who died before their infection was confirmed microbiologically, and 126 patients (16%) were not followed up for a full 30 days after hospitalization. However, neither the exclusion rates before microbiological confirmation (14/369 vs 16/447, *P* = 0.98) nor the rates of loss to follow-up within 30 days of hospitalization (104/353 vs 127/418, *P* = 0.84) differed between the MRSA and MSSA groups. Fourth, our results may not convey a complete picture of the disease in the community because all the participating centers were secondary or tertiary hospitals. Fifth, we did not evaluate a seasonal variation in *S. aureus* infections because the study period was only 8 months long. Finally, we did not analyze the genetic background of the MRSA isolates. The antibiotic susceptibility profiles of the CA-MRSA and HA-MRSA isolates for non-beta-lactams were significantly different (Fig. 2). However, it was not possible to infer potential common genetic types of MRSA from their profiles because the most common nosocomial MRSA strains such as ST5 and ST239 are also circulating in the Korean community [5]. Despite these limitations, the study has definite strengths, such as its large-scale, prospective, and nationwide design.

In conclusion, CA-MRSA cases have increased in Korea since the last nationwide survey in 2005. The risk of MRSA infection should be considered where there has been recent exposure to 3rd generation cephalosporins, or close contact with chronically-ill patients. We do not recommend universal empirical anti-MRSA treatment in patients presenting with invasive CO-*S. aureus* infections in Korea if there are no risk factors for methicillin resistance. Clinicians should consider empirical anti-MRSA treatment only in the clinical setting of severe illness.

## Supporting Information

S1 Table Univariate analysis of risk factors for methicillin-resistant *Staphylococcus aureus* (MRSA) infections in 464 Korean patients with community-onset healthcare-associated (HA) invasive *S. aureus* infections.
(DOCX)

S2 Table Multivariate analysis of risk factors for methicillin-resistant *Staphylococcus aureus* (MRSA) infection in 464 patients with community-onset healthcare-associated invasive *S. aureus* infections.
(DOCX)
