# Supplementary material for: Clinical and Epidemiological Factors Associated with Methicillin Resistance in Community-Onset Invasive Staphylococcus aureus Infections: Prospective Multicenter Cross-Sectional Study in Korea
Source: PLoS One. 2014 Dec 8;9(12):e114127. doi: 10.1371/journal.pone.0114127 (PMC4259386; doi:10.1371/journal.pone.0114127)
Supplement: S1 Table — Univariate analysis of risk factors for methicillin-resistant Staphylococcus aureus (MRSA) infections in 464 Korean patients with community-onset healthcare-associated (HA) invasive S. aureus infections. (DOCX) [file pone.0114127.s001.docx]

**Table S1.** Univariate analysis of risk factors for methicillin-resistant *Staphylococcus aureus* (MRSA) infections in 464 Korean patients with community-onset healthcare-associated (HA) invasive *S. aureus* infections.

| Characteristics | HA-MSSA (n=211) | HA-MRSA (n=253) | *P* Value | OR (95% CI) |
| --- | --- | --- | --- | --- |
| Male gender | 130 (61.6%) | 151 (59.7%) | 0.672 | 1.084 (0.746-1.576) |
| Age, years (mean ± SD) | 59.2 ± 19.4 | 61.2 ± 21.2 | 0.292 |  |
| Age (per 10-year increase) |  |  | 0.292 | 1.049 (0.959-1.148) |
| Age group |  |  |  |  |
| ≤15 yr | 9 (4.3%) | 18 (7.1%) | 0.192 | 1.756 (0.756-3.911) |
| 16-64 yr | 106 (50.2%) | 90 (35.6%) | 0.001 | **0.547 (0.377-0.794)** |
| ≥65 yr | 96 (45.5%) | 145 (57.3%) | 0.011 | **1.608 (1.113-2.324)** |
| Type of infection |  |  |  |  |
| Primary bacteremia | 27 (12.8%) | 32 (12.6%) | 0.962 | 0.987 (0.570-1.707) |
| CLA-BSI | 20 (9.5%) | 20 (7.9%) | 0.548 | 0.820 (0.429-1.568) |
| Pneumonia | 20 (9.5%) | 51 (20.2%) | 0.001 | **2.411 (1.386-4.194)** |
| SSTI | 43 (20.4%) | 30 (11.9%) | 0.012 | **0.526 (0.316-0.873)** |
| SSI | 18 (8.5%) | 26 (10.3%) | 0.523 | 1.228 (0.654-2.308) |
| BJI | 44 (20.9%) | 39 (15.4%) | 0.128 | 0.692 (0.430-1.114) |
| IAI | 20 (9.5%) | 22 (8.7%) | 0.770 | 0.910 (0.482-1.717) |
| Vascular infection | 8 (3.8%) | 15 (5.9%) | 0.291 | 1.599 (0.665-3.849) |
| Presence of comorbidities | 168 (79.6%) | 204 (80.6%) | 0.786 | 1.066 (0.674-1.684) |
| Charlson index (mean ± SD) | 2.5 ± 2.3 | 2.5 ± 2.2 | 0.885 |  |
| Charlson index (per 1 unit increase) |  |  | 0.884 | 1.006 (0.927-1.092) |
| Previous admission (<1 yr) | 171 (81.0%) | 204 (80.6%) | 0.911 | 0.974 (0.612-1.550) |
| Residence in a long-term care facility (<1 yr) | 20 (9.5%) | 44 (17.4%) | 0.014 | **2.011 (1.144-3.533)** |
| History of dialysis (<1 yr) | 34 (16.1%) | 43 (17.0%) | 0.799 | 1.066 (0.652-1.744) |
| History of SSI (<1 m) | 11 (5.2%) | 23 (9.1%) | 0.110 | 1.818 (0.865-3.823) |
| Presence of percutaneous devices | 37 (17.5%) | 50 (19.8%) | 0.540 | 1.158 (0.723-1.855) |
| Previous MRSA carriage (<1 yr) | 8 (3.8%) | 44 (17.4%) | <0.001 | **5.342 (2.455-11.627)** |
| Previous antibiotic exposure (<6 m) |  |  |  |  |
| Any | 89 (42.2%) | 154 (60.9%) | <0.001 | **2.132 (1.470-3.093)** |
| Cephalosporins | 56 (26.5%) | 90 (35.6%) | 0.037 | **1.528 (1.025-2.279)** |
| 1st generation cephalosporins | 18 (8.5%) | 20 (7.9%) | 0.807 | 0.920 (0.473-1.789) |
| 3rd generation cephalosporins | 31 (14.7%) | 67 (26.5%) | 0.002 | **2.092 (1.304-3.355)** |
| Quinolones | 15 (7.1%) | 35 (13.8%) | 0.020 | **2.098 (1.112-3.958)** |
| Immunosuppressant use (<1 yr) | 21 (10.0%) | 20 (7.9%) | 0.439 | 0.777 (0.409-1.475) |
| Steroid use | 6 (2.8%) | 9 (3.6%) | 0.665 | 1.260 (0.441-3.600) |
| History of acupuncture (<1 m) | 11 (5.2%) | 7 (2.8%) | 0.174 | 0.517 (0.197-1.359) |
| History of OPD visit (<1 yr)^a^ | 164 (78.5%) | 195 (77.7%) | 0.841 | 0.955 (0.613-1.489) |
| Close contact with HCWs (<1 m) | 75 (35.5%) | 113 (44.7%) | 0.046 | **1.464 (1.006-2.130)** |
| Close contact with chronically-ill patients (<1 m) | 7 (3.3%) | 22 (8.7%) | 0.017 | **2.776 (1.162-6.632)** |
| Living with children ≤5 years old^b^ | 30 (21.9%) | 48 (25.4%) | 0.465 | 1.214 (0.721-2.044) |
| Current smoking | 20 (9.5%) | 21 (8.3%) | 0.656 | 0.864 (0.455-1.642) |
| Alcohol intake | 38 (18.0%) | 40 (15.8%) | 0.528 | 0.855 (0.525-1.392) |
| Communal living | 8 (3.8%) | 20 (7.9%) | 0.064 | **2.178 (0.939-5.052)** |

Data are numbers (%) of patients unless stated otherwise. Values in bold indicate statistically significant factors (*P*<0.10). MSSA, methicillin-susceptible *S. aureus*; SD, standard deviation; CLA-BSI, central line-associated blood stream infection; SSTI, skin and soft tissue infection; SSI, surgical site infection; BJI, bone and joint infection; IAI, intra-abdominal infection; OPD, outpatient department; HCWs, healthcare workers.

^a^Data on OPD visits for 4 patients were censored, and 460 patients (209 and 251 patients in the MSSA and MRSA groups, respectively) were included in the analysis.

^b^Data on 138 patients living with children ≤5 years old were censored, and 326 patients (137 and 189 patients in the MSSA and MRSA groups, respectively) were included in the analysis.
