# Supplementary material for: Clinical and Epidemiological Factors Associated with Methicillin Resistance in Community-Onset Invasive Staphylococcus aureus Infections: Prospective Multicenter Cross-Sectional Study in Korea
Source: PLoS One. 2014 Dec 8;9(12):e114127. doi: 10.1371/journal.pone.0114127 (PMC4259386; doi:10.1371/journal.pone.0114127)
Supplement: S2 Table — Multivariate analysis of risk factors for methicillin-resistant Staphylococcus aureus (MRSA) infection in 464 patients with community-onset healthcare-associated invasive S. aureus infections. (DOCX) [file pone.0114127.s002.docx]

**Table S2.** Multivariate analysis of risk factors for methicillin-resistant *Staphylococcus aureus* (MRSA) infection in 464 patients with community-onset healthcare-associated invasive *S. aureus* infections

| Characteristics | *P Value* | Adjusted OR (95% CI) |
| --- | --- | --- |
| Age group of 16-64 year | 0.027 | 0.364 (0.149-0.890) |
| Presentation as pneumonia | 0.029 | 1.975 (1.073-3.633) |
| Previous MRSA carriage (<1 yr) | <0.001 | 5.327 (2.371-11.970) |
| Previous use of 3rd generation cephalosporins (<6 m) | 0.037 | 1.726 (1.034-2.882) |
| Close contact with chronically-ill patients (<1 m) | 0.027 | 2.828 (1.129-7.084) |
